# Effects of percutaneous cryoablation for renal tumor on overall and split renal function

**DOI:** 10.1007/s11604-024-01589-1

**Published:** 2024-05-15

**Authors:** Takuji Yamagami, Rika Yoshimatsu, Noriko Nitta, Kana Miyatake, Hitomi Iwasa, Junki Shibata, Marina Osaki, Hitomi Maeda, Yoshihiro Noda, Tomoaki Yamanishi, Tomohiro Matsumoto, Shinkuro Yamamoto, Takashi Karashima, Keiji Inoue

**Affiliations:** 1 Department of Diagnostic and Interventional Radiology, Kochi Medical School, Kochi University, Kohasu, Oko-Cho, Nankoku, Kochi, 783-8505 Japan; 2 Department of Radiology, Kochi Health Sciences Center, 2125-1 Ike, Kochi, Japan; 3 Department of Urology, Kochi Medical School, Kochi University, Kohasu, Oko-Cho, Nankoku, Kochi, 783-8505 Japan

**Keywords:** Cryoablation, Mercaptoacetyltriglycine, Renal functions, Renal tumor

## Abstract

**Purpose:**

To evaluate retrospectively the influence of percutaneous cryoablation for small renal tumors on total and affected kidney function and risk factors associated with worsening function of the affected kidney.

**Materials and methods:**

Between April 2016 and March 2022, 27 patients who underwent cryoablation for small renal tumors at our institution participated in this study, which investigated time-dependent changes in postoperative renal function. We evaluated estimated glomerular filtration rates (eGFRs) and split renal function revealed by scintigraphy using 99 m technetium-mercaptoacetyltriglycine (99mTc-MAG3) before cryoablation and at 1 week, 1 month, and 6 months after cryoablation. Numerous variables were analyzed to assess risk factors for worsening renal function.

**Results:**

Baseline eGFR (mean ± standard deviation) was 56.5 ± 23.7 mL/min/1.73 m^2^ (mean ± SD; range, 20.5–112.5). Mean eGFRs at 1 week, 1 month, and 6 months after cryoablation were 57.4 ± 24.5 (19.1–114.9), 57.1 ± 25.1 (21.5–114.9), and 53.8 ± 23.9 mL/min/1.73 m^2^ (20.0–107.5), respectively. Changes were statistically insignificant (*p* = 1.0000, = 0.6749, and = 0.0761, respectively). Regarding split renal function, mean baseline contribution of the affected kidney determined by 99mTc-MAG3 was 49.7% ± 6.0% (38.8–63.3%); these rates at 1 week, 1 month, and 6 months after cryoablation were 43.7% ± 8.8 (29.1–70.6%), 46.2% ± 7.7% (32.6–70.3%), and 46.0% ± 8.5% (32.5–67.6%), respectively. Differences from baseline were significant for all periods (*p* < 0001, < 0001, = 0.0001, respectively). Serum C reactive protein and lactate dehydrogenase at 1 day following cryoablation, tumor’s nearness to the collecting system or sinus, and volume of ablated normal renal parenchyma were significantly correlated with decreased contributions of the affected kidney by > 10% after cryoablation.

**Conclusion:**

Unlike total renal function, affected kidney function could worsen after cryoablation.

## Introduction

Renal cryoablation has been accepted as a therapeutic option for small renal tumors, with an increasing number of reports focusing on this procedure [1–4]. Among them have been reports evaluating post-procedural renal function [2, 3, 5–7]. These studies evaluated renal function after cryoablation therapy according to changes in serum creatinine or creatinine clearance. While total renal function can be evaluated by monitoring serum creatinine or creatinine clearance, changes in the function of the affected kidney cannot be determined by this means because of the influence of the contralateral kidney. A previous report [8] involving scintigraphy using 99 m technetium-mercaptoacetyltriglycine (99mTc-MAG3) suggested that renal function of the affected kidney decreased early after the procedure, but could eventually begin to recover. However, that previous study only included nine patients; hence, further study with a larger number of patients is required to draw conclusions on split renal function after cryoablation.

In the present study, we evaluated changes in total renal function before and after cryoablation therapy, as well as function of the affected kidney by assessment of split renal function with scintigraphy using 99mTc-MAG3 in a larger number of participants than previously studied. In addition, risk factors associated with worsening of split renal function were investigated.

## Materials and methods

Approval for this retrospective study was obtained from the institutional ethics committee (ERB-109897).

### Patients

Between March 2016 and December 2022, 73 sessions of cryoablation were performed for small renal tumors in 49 patients at our institution (Table 1). Of those patients, 27 who satisfied the study admission criteria were included in the study (Table 2). Inclusion criteria were: (1) Not having received more than two cryoablation sessions at the same site during the study period; (2) Not undergoing renal dialysis; (3) Estimated glomerular filtration rate (eGFR) > 15 mL/min/1.73m^2^; (4) Having two functioning kidneys; (5) Having a single renal tumor; and (6) Evaluation of serum creatinine and creatinine clearance and performance of scintigraphy using 99mTc-MAG3 before and after cryoablation. The mean age of the patients was 74.2 years (range: 37–90 years, median: 78 years), and mean tumor size was 24.7 ± 7.2 mm (range: 11.8–36.7, median: 26.2 mm). All 27 patients underwent percutaneous needle biopsy before cryoablation and, with the exception of one patient, renal cell carcinoma was confirmed. The tissue specimen obtained from that one patient was inadequate; nevertheless, renal cell carcinoma was highly suspected by diagnostic imaging including dynamic enhanced computed tomography (CT). Scintigraphy using 99mTc-MAG3 before cryoablation was conducted before and after biopsy in 21 and six patients, respectively. Previous history of other local treatments showed that partial nephrectomy had been performed in six patients (ipsilateral, *n* = 2; contralateral, *n* = 2; bilateral, *n* = 2), while selective arterial embolization for bleeding angiomyolipoma of the contralateral site had been performed in one patient.

**Table 1:** Patients who underwent cryoablation

Inclusion in the study	Number of patients
Yes	27
No	22
Exclusion criteria	
Not having received > 2 cryoablation	16^*,**,***^
After total nephrectomy of contralateral kidney	6^*,**^
A single functioning kidney	2
Cryoablation performed for two tumors on the same day	1^**^
Estimated glomerular filtration rate < 15 mL/min/1.73 m^2^	1
Scintigraphy using 99mTc-MAG3 not performed before cryoablation	1
Undergoing renal dialysis	1^***^

^*^Overlapping in 3

^**^Overlapping in 1

^***^Overlapping in 1

*99mTc-MAG3*: 99 m technetium-mercaptoacetyltriglycine

**Table 2:** Characteristics of study participants with and without occurrence of split renal dysfunction after cryoablation

	Decrease in split renal function				*P *value
	No (*n* = 22)	*n*	Yes (*n* = 5)	*n*	
Participant characteristic					
Sex					0.628
Female	9 (41%)		3 (60%)		
Male	13 (59%)		2 (40%)		
Age (years)	73.1 ± 13.9 (37–90)		79.2 ± 9.8 (63–81)		0.3637
Lesion factor					
Tumor size (mm)	24.1 ± 7.9 (11.8–36.7)		27.5 ± 3.0 (24.2–32.3)		0.3639
Nearness to collecting system or sinus (mm)	4.8 ± 4.6 (0–12.8)		0		0.0317
Exophytic/endophytic properties					0.517
≥ 50%		7		1	
< 50%		12		4	
Entirely		3		0	
Procedure-related factors					
Performance of lipiodol marking					0.4735
Yes	20 (91%)		4 (80%)		
No	2 (9%)		1 (20%)		
Number of cryoprobes inserted	3 ± 0.8		3.8 ± 0.8		0.06
2		7		0	
3		8		2	
4		7		2	
5		0		1	
Ablation-causing factor					
CRP day after cryoablation (mg/dL)	1.8 ± 1.1 (0.19–3.63)	22	6.7 ± 6.0 (0.77–14.77)	4	0.0008
LDH day after cryoablation (mg/dL)	532.5 ± 184.4 (215–777)	22	811.5 ± 404.7 (344–1,301)	4	0.0311
Volume of ablated normal renal parenchyma (mL)	14.1 ± 7.5 (4.523–33.149)	21	26.3 ± 15.2 (6.244–44.274)	5	0.0141
Occurrence of complication		15*		3**	0.7261

Data are presented as the mean ± standard deviation (range) or number (%), unless otherwise indicated

^*^Hemorrhage alone, *n* = 8; hematuria alone, *n* = 6; both, *n* = 1

^**^Hemorrhage alone, *n* = 2; hematuria alone, *n* = 1

*CRP* C reactive protein, *LDH* lactate dehydrogenase

### Procedures

After patients provided written informed consent, the ablation procedure was performed by one of four interventional radiologists in our institution who is experienced in ablation procedures, such as radiofrequency ablation and cryoablation. All procedures were performed using an interventional CT system that included a unified CT and angiography unit (Aquilion LB combined with Infinix Celeve-i INFX-8000 V) provided by Canon Medical Systems (Ohtawara, Japan). In 24 of the 27 sessions, lipiodol marking was performed; iodized oil (Lipiodol Ultrafluid; Guerbet Japan, Tokyo, Japan) was selectively infused into the tumor-feeding arteries, followed by the addition of a small amount of gelatin particles measuring 1–2 mm in diameter to clearly visualize the tumor on CT at the time of cryoablation [9]. In all 24 sessions, scintigraphy using 99mTc-MAG3 was performed before lipiodol marking. Cryoablation of the renal tumor was performed within 1 week after lipiodol marking.

The cryoablation procedure was conducted with a CryoHit device (Boston Scientific Japan, Tokyo, Japan). Based on the tumor diameter, 2–5 (median: 3) IceSeed or IceRod needles (Boston Scientific Japan) were used. With the patient in the prone or lateral position and under local anesthesia, cryoprobes were inserted percutaneously under CT fluoroscopic guidance (Aquilion LB; Canon Medical Systems) to the site of the tumor. Cryoablation was performed in two cycles of a 10-min freeze and 5-min thaw. The ice ball was defined as a low-density area that surrounded the cryoprobes during freezing. CT images were obtained at the end of both freezing times to assess the ice ball. If the tumor was not covered with an ice ball, additional cryoprobes were placed to cover the tumor entirely, and additional cycles of 10-min freeze and 5-min thaw were performed. Enhanced dynamic CT was obtained approximately 1 week after cryoablation to evaluate therapeutic effects and the presence of any complication in all patients, with one exception in which plain CT was conducted. A 320-detector row CT (AquilionONE; Canon Medical Systems) was used for both enhanced dynamic and plain CT.

In 15 cases, a critical structure such as the colon (*n* = 7), liver (*n* = 3), duodenum (*n* = 2), psoas (*n* = 2), small intestine (*n* = 1), or pancreas (n = 1) was located adjacent to the renal tumor to be ablated. In these cases, hydrodissection was performed to prevent injury of organs adjacent to renal tumors [10]. In three cases with the ureter running near the renal tumor, retrograde pyeloperfusion of warm saline (38–40 °C) via a ureteric catheter was conducted [10] to avoid damage to the ureter. In another three cases with tumors located at the upper pole of the kidney and necessitating a transthoracic approach, a small pneumothorax was artificially induced immediately before insertion of the cryoprobe [11]. Details of these procedures were described elsewhere [10, 11].

### Analysis of renal function

The eGFR was examined before and approximately 1 week, 1 month (mean ± standard deviation [SD]: 41 ± 10 days, range: 29–55 days, median: 41 days), and 6 months (mean ± SD: 191 ± 61 days, range: 118–344 days, median: 178 days) after cryoablation. Scintigraphy using 99mTc-MAG3 was also performed before cryoablation and approximately 1 week, 1 month (mean ± SD: 43 ± 10 days, range: 29–83 days, median: 41 days), and 6 months (mean ± SD: 176 ± 51 days, range: 118–352 days, median: 164 days) after cryoablation. All 99mTc-MAG3 scintigraphy data were acquired using a SPECT CT system (Symbia T2 scanner; Siemens, Munich, Germany). Immediately after bolus injection of 300 MBq of 99mTc-MAG3, first pass images (1 frame/1 s for 1 min) and dynamic renal images (1 frame/10 s for 24 min) were recorded, and data were processed. Total renal function was evaluated by the eGFR, and the function of each kidney was assessed separately with split renal function testing performed by calculating MAG3 clearance.

### Investigated parameters

**Fig. 1:**
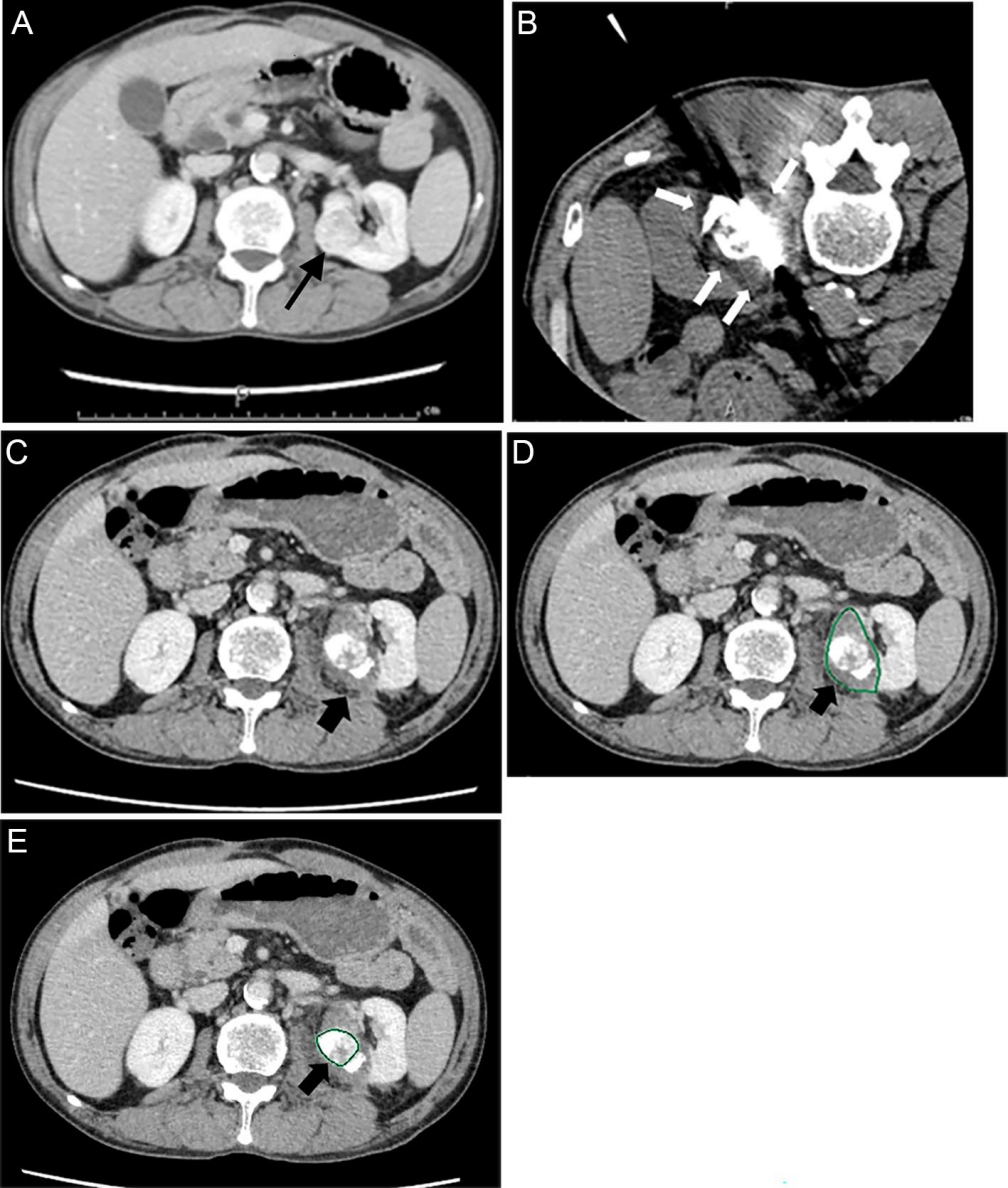
A 63-year-old woman with renal cell carcinoma underwent percutaneous cryoablation under computed tomography (CT) guidance. **a** Enhanced CT before cryoablation showing a renal tumor (size: 26 mm) (arrow). Tumor was attached to the renal hilum (nearness to the collecting system or sinus was 0 mm) and the exophytic/endophytic property was < 50%. **b** CT obtained at the end of the cryoablation procedure showing the cryoprobe penetrating the tumor with accumulation of lipiodol infused at the time of marking and surrounded by an ice ball (white arrows). Note: three cryoprobes were inserted into the tumor. **c** Enhanced CT obtained 5 days after cryoablation showing that the tumor was sufficiently surrounded by a poorly enhanced area (arrow). **d** Periphery of the poorly enhanced area in the renal parenchyma on one axial image manually drawn with the drawing tool on a three-dimensional (3D) workstation (arrow). The volume of the unenhanced area inside the renal parenchyma plus the tumor over the contiguous transaxial images was measured (35.66 mL). **e** Periphery of the tumor manually drawn with the drawing tool on a 3D workstation (arrow). Volume of the tumor: 8.84 mL. By subtracting this value from the volume measured in **d**, the volume of ablated normal renal parenchyma was calculated as 26.82 mL

Investigated parameters were time-dependent changes in eGFR and the contribution of the affected kidney to renal function detected by scintigraphy using 99mTc-MAG3 before and after cryoablation. We also investigated risk factors for worsening of split renal function. In this study, decreased split renal function was defined as decreases by > 10% in the contribution of the affected kidney to the total renal function on scintigraphy with 99mTc-MAG3 compared with pre-ablation values in at least one period after cryoablation. Risk factors investigated in this study included patients’ sex and age, serum C reactive protein (CRP) and lactate dehydrogenase (LDH) levels the day following cryoablation, whether lipiodol marking was or was not performed before cryoablation, tumor size, number of cryoprobes used, nearness to the collecting system or sinus, exophytic/endophytic properties (i.e., ≥ 50%,  < 50%, or entirely endophytic), volume of ablated normal renal parenchyma, and occurrence of complications. Definitions of nearness to the collecting system or sinus and exophytic/endophytic properties have been described previously [12]. Volume of ablated normal renal parenchyma was calculated as follows. Contiguous transaxial images (thickness: 5 mm) were reconstructed from the volumetric data set on the late phase of the enhanced dynamic CT obtained 1 week after cryoablation. The poorly enhanced area of renal parenchyma surrounding the tumor was manually drawn on each slice using the drawing tool on a three-dimensional (3D) workstation (SYNAPSE VINCENT; Fuji Film Medicals, Tokyo, Japan). Thereafter, software (3D viewer; Fuji Film Medicals) was used to measure the volume of the poorly enhanced renal parenchyma surrounding the tumor, which was deemed the volume of ablated normal renal parenchyma. In cases in which drawing only the poorly enhanced area of renal parenchyma surrounding the tumor was difficult, we first measured the poorly enhanced area inside the renal parenchyma plus the tumor and subsequently measured the tumor only. Finally, we calculated the volume of the poorly enhanced renal parenchyma surrounding the tumor by subtracting the latter value from the former value (Fig. 1).

### Statistical analysis

The difference in eGFR before and after cryoablation and split renal function by calculating MAG3 clearance before and after cryoablation were assessed by the paired* t *test. Regarding risk factors, quantitative variables were compared using Student’s *t* test, while qualitative variables were compared using Fisher’s exact test or Pearson’s chi-square test. Stepwise multivariate logistic regression analysis was also performed. A *p *value < 0.05 was considered to indicate a statistically significant difference. For statistical analysis, commercial software (JMP 14; SAS Japan, Tokyo, Japan) was used.

## Results

Tumors were ablated successfully without any residual tumor tissue in all 27 patients. Complications related to cryoablation were hemorrhage surrounding the ablated tumors shown on CT obtained immediately after cryoablation (*n* = 11) and hematuria (*n* = 8). In one patient, both events occurred. All complications were minor according to the classification established by the Society of Interventional Radiology [13, 14].

**Fig. 2:**
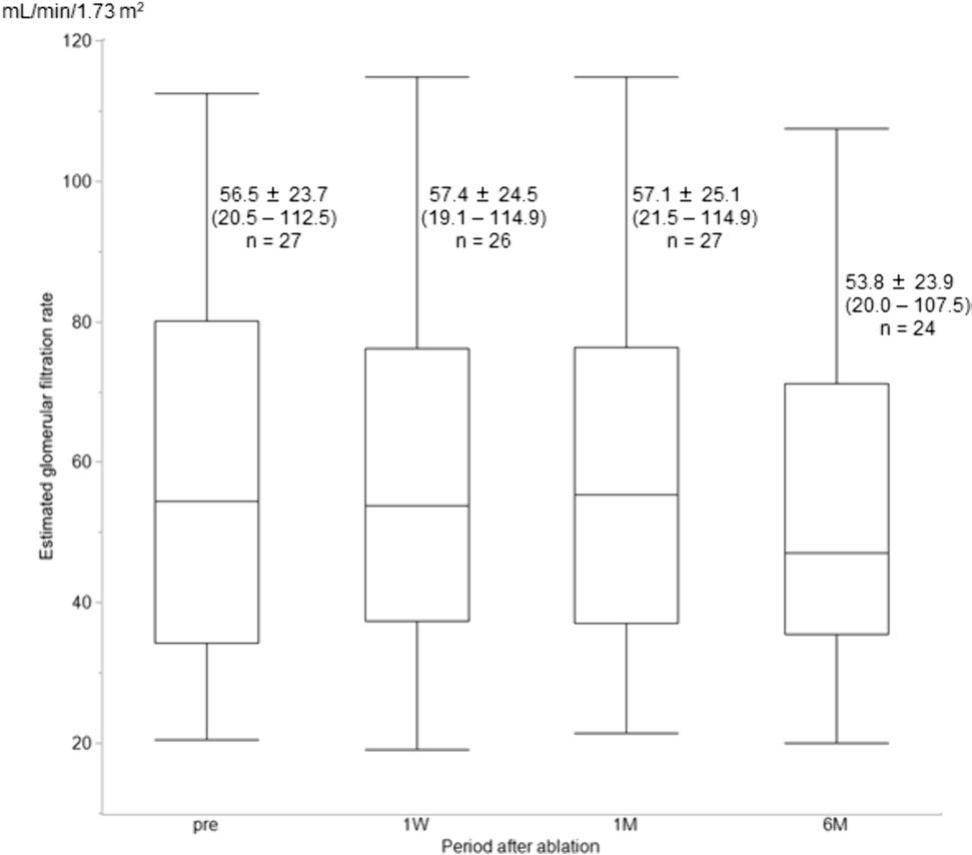
Changes in estimated glomerular filtration rate before and after cryoablation

The eGFR (mean ± SD) changed from 56.47 ± 23.67 mL/min/1.73 m^2^ (range: 20.5–112.5; *n* = 27) to 57.35 ± 24.54 mL/min/1.73 m^2^ (range: 19.1–114.9; *n* = 26), 57.14 ± 25.08 mL/min/1.73 m^2^ (range: 21.5–114.9; *n* = 27), and 53.77 ± 23.86 mL/min/1.73 m^2^ (range: 20–107.5; *n* = 24) at 1 week, 1 months, and 6 months after cryoablation, respectively (Fig. 2). These changes were not statistically significant (*p* = 1.000, 0.6749, and 0.0761, respectively). Compared with pre-ablation values, the eGFR was higher in 38.5% (10/26), 44.4% (12/27), and 29.2% (7/24) of the participants for whom the eGFR was evaluated at 1 week, 1 month, and 6 months after cryoablation, respectively.

**Fig. 3:**
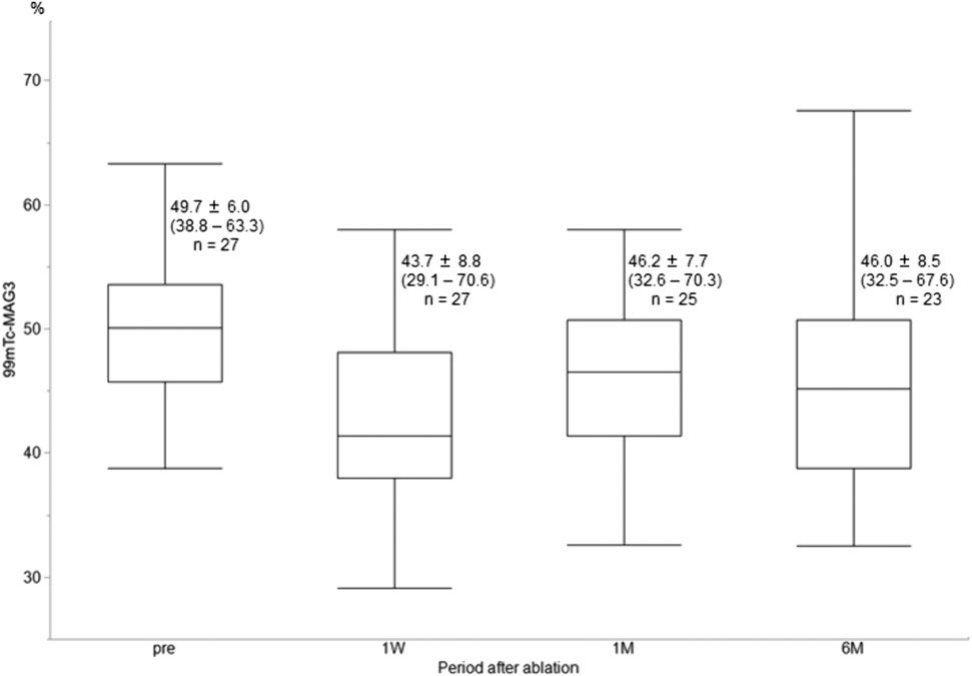
Changes in the contribution of the affected kidney by calculating 99 m technetium-mercaptoacetyltriglycine before and after cryoablation

With regard to split renal function, the contribution of the affected kidney decreased from a mean ± SD of 49.71% ± 6.03% (range: 38.8–63.3%; *n* = 27) to 43.74% ± 8.76% (range: 29.1–70.6%; *n* = 27), 46.20% ± 7.72% (range: 32.6–70.3%; *n* = 25), and 45.96% ± 8.48% (range: 32.5–67.6%; *n* = 23) at 1 week, 1 month, and 6 months after cryoablation, respectively (Fig. 3); all decreases were statistically significant (*p* < 0.0001,  < 0.0001, and = 0.0001, respectively). Decreases compared with precryoablation were noted in 92.6% (25/27), 88.0% (22/25), and 82.6% (19/23) of the participants at 1 week, 1 month, and 6 months after cryoablation, respectively.

In five cases, the contribution of the affected kidney to total renal function on scintigraphy using 99mTc-MAG3 decreased by > 10% in any period after cryoablation compared with precryoablation values (Table 3). Serum CRP and LDH levels the day following cryoablation, nearness to the collecting system or sinus, and volume of ablated normal renal parenchyma were significantly correlated with decreases in contributions of the affected kidney > 10% after cryoablation (CRP level, *p* = 0.0008, Student’s *t* test; LDH level, *p* = 0.0311, Student’s *t*-test; nearness to collecting system or sinus, *p* = 0.0317, Student’s *t*-test; volume of ablated normal renal parenchyma, *p* = 0.0141, Student’s *t* test). In contrast, sex (*p* = 0.628, Fisher’s exact test), age (*p* = 0.3637, Student’s *t* test), performance of lipiodol marking (*p* = 0.4735, chi*-*square test), tumor size (*p* = 0.3639*,* Student’s *t* test), number of cryoprobes used (*p* = 0.0600, Student’s *t*-test), and exophytic/endophytic properties (*p* = 0.5170, Pearson’s chi-square test), and occurrence of complication (*p* = 0.7261, Pearson’s chi-square test) were not significantly associated with decreases by > 10% in contributions of the affected kidney to total renal function. Stepwise multivariate logistic regression analysis revealed significant differences between groups in terms of CRP levels the day following cryoablation (*p* = 0.0040) and volume of ablated normal renal parenchyma (*p* = 0.0371).

**Table 3:** Decreases in 99mTc-MAG3 > 10% in each period after cryoablation compared with pre-cryoablation

Patient number	1	2	3	4	5
Sex	Male	Female	Female	Male	Female
Age, years	63	79	81	89	84
Previous history of local treatment for renal diseases	No	No	No	No	No
Size of tumor, mm	26	24.2	26.7	32.3	28.1
Nearness to collecting system or sinus, mm	0	0	0	0	0
Exophytic/endophytic properties	< 50	< 50	< 50	< 50	≥ 50
Performance of lipiodol marking	Yes	Yes	Yes	Yes	No
Number of cryo-needles used	3	3	5	4	4
Complications related to cryoablation	Hemorrhage	Hematuria	Hemorrhage	None	None
CRP level at 1 day after cryoablation, mg/dL	0.77	NA	3.92	14.77	7.49
LDH level at 1 day after cryoablation, U/L	672	NA	1301	929	344
Volume of ablated normal renal parenchyma, mL	26.8	17	44.3	37	6.2
eGFR, mL/min/1.73 m^2^					
Prior to cryoablation	80.9	54.5	83.1	31.8	22.5
1 week after cryoablation	77.5	48.9	66.2	38.8	24
1 month after cryoablation	81.8	54.5	66.2	37.3	24.1
6 months after cryoablation	73.1	43.3	81.3	40.1	25.9
Contribution of the affected kidney on 99mTc-MAG3, %					
Prior to cryoablation	50.2	47.2	50.1	45.7	50
1 week after cryoablation	37.4*	33.5*	38*	31.9*	39.1
1 month after cryoablation	41.8*	42.8	41*	32.6*	42.6*
6 months after cryoablation	43*	38.3*	NA	33.8*	42.9*

*99mTc-MAG3* 99 m technetium-mercaptoacetyltriglycine, *CRP* C reactive protein, *eGFR* estimated glomerular filtration rate, *LDH* lactate dehydrogenase

^*^Period in which the contribution of the affected kidney decreased by > 10% compared with that recorded prior to cryoablation

## Discussion

Although partial or radical nephrectomy is the standard clinical therapy for small renal tumors, reports of ablation therapies, including cryoablation, have been increasing [1, 4]. Cryoablation can be an attractive alternative for patients of advanced age or with significant comorbidities who prefer a proactive approach but are not considered good candidates for surgery [1, 2].

From the viewpoint of minimizing renal dysfunction, cryoablation may be advantageous compared with nephrectomy [1, 15, 16]. Changes in renal function after cryoablation have been previously evaluated [2, 3, 5–8]. In those studies, serum creatinine or creatinine clearance was the most frequently used parameter to evaluate renal function. In the majority of previous reports [2, 5, 6, 8], cryoablation had a minimal impact on worsening renal function. In addition, there was no significant decrease in renal function noted in cases in which selective transarterial embolization was performed before cryoablation [1, 8, 9, 17, 18]. However, a few reports have demonstrated worsening of renal function after cryoablation. According to Malcolm et al. [7], de novo chronic kidney disease was observed in 11% of study participants after cryoablation over a mean period of 30 months. Tsivian et al. [3] reported that eGFR decreased by approximately 5 mL/min/1.73 m^2^ over a 2-year period after cryoablation. That decline was similar between patients with and without pre-existing renal insufficiency. Of note, even in reports of a negative impact of cryoablation on postoperative renal function, severity appeared to be mild [3, 7]. Although the abovementioned reports evaluated total renal function, they did not evaluate split renal function [1–3, 5–7, 17]. However, a single previous study did evaluate split renal function [8].

Similarly, in the present study, the eGFR did not significantly change at any period after cryoablation. However, evaluation of split renal function showed that the contribution of the affected kidney decreased significantly in all three periods after cryoablation, although the proportion of patients with reduced split renal function in the affected kidney decreased slightly. This finding is slightly over time after ablation different from the results of the abovementioned previous study [8]. Decreases occurred in 92.6%, 88.0%, and 82.6% of the participants at 1 week, 1 month, and 6 months after cryoablation, respectively.

Focusing on five cases in which the contribution of the affected kidney to total renal function on scintigraphy with 99mTc-MAG3 was markedly decreased (i.e., by > 10% in at least one period after cryoablation), the risk factor appeared to be related to CRP and LDH values the day after cryoablation. CRP is a well-established marker of inflammation, with levels rising in the presence of inflammation [19]. LDH is an enzyme in serum that works when glucose is converted into energy. It is present in several organs, including the kidney. Following the occurrence of abnormalities or injury in such organs, LDH can enter the bloodstream, leading to high levels [20]. Thus, care must be taken regarding renal dysfunction in cases of severe inflammatory reactions or large areas of organ breakdown after cryoablation according to imaging modalities, such as CT. It was revealed that cryoablation near the collecting system or sinus was a risk factor for worsening of split renal function. Among the five patients in whom the contribution of the affected kidney to the total renal function on scintigraphy with 99mTc-MAG3 was markedly decreased (i.e., by > 10% after cryoablation), the tumor was in or touched the collecting system or sinus. The present findings also revealed that, in cases in which the volume of ablated normal renal parenchyma was large, dysfunction of the affected kidney would occur after cryoablation with a statistically significant high possibility. This led us to suggest that physicians should take care to minimize the volume of ablated normal renal parenchyma within a range that ensures a sufficient margin [1]. This is especially important when supplementation of renal function by a healthy kidney cannot be expected either due to the existence of a single kidney or a non-functioning second kidney.

The limitation of this retrospective and observational study mainly lies in its relatively small number of patients; however, the sample size was larger than that included in a previous similar study [8]. In addition, patients who underwent percutaneous biopsy and/or lipiodol marking prior to scintigram were included, although the amount and distribution of the embolic agent used at the time of lipiodol marking was minimal. We cannot conclude with certainty that these procedures would have no influence on renal function. Nonetheless, such an influence would be slight because the statistical analysis showed that the lipiodol marking was not significantly related to worsening of split renal function as revealed by the present study. Another limitation would be that local treatments (such as partial nephrectomy) had been previously performed in a few patients. However, worsening of split renal function by > 10% compared with pretreatment did not occur in any of these patients, suggesting that the influence of such treatments on split renal dysfunction might have been minimal. If possible, a prospective study of split renal function in a multicenter setting with a larger number of patients, more rigorous protocol, and longer follow-up period is desirable. However, thus far, there has been only one report of changes in split renal function after cryoablation [8]. From this perspective, we think the present study is of value.

Total renal function is important in clinical practice because supplementation of renal function by a healthy kidney can be expected irrespective of the degree of damage to the affected side caused by cryoablation. However, there are several situations in which a healthy kidney would be damaged, such as an accident, metastasis to the contralateral kidney, etc. Hence, the occurrence of a decrease in function of the affected kidney suggests that care is necessary to minimize worsening renal function of the affected side after cryoablation and to avoid careless damage of the unaffected kidney, in the follow-up period after ablation. This is particularly important in patients with the four risk factors revealed in this study. Moreover, the results of our study may be important in considering the use of cryoablation for patients with a single kidney.

In conclusion, although total renal function could be maintained after cryoablation, there is a possibility that dysfunction of the affected kidney might occur after cryoablation and continue over time. Worsening of function of the affected kidney can occur, in particular when serum CRP or LDH levels rise greatly after cryoablation, as well as when the tumor is located at the renal hilum or the volume of ablated normal renal parenchyma is large.
